# Gut microbiome: a new player in gastrointestinal disease

**DOI:** 10.1007/s00428-017-2277-x

**Published:** 2017-12-14

**Authors:** Gregor Gorkiewicz, Alexander Moschen

**Affiliations:** 1 Institute of Pathology, Medical University of Graz, Neue Stiftingtalstrasse 6, 8010 Graz, Austria; 2 Christian Doppler Laboratory for Mucosal Immunology & Division of Internal Medicine I, Department of Medicine, Medical University Innsbruck, Peter-Mayr-Strasse 1, 6020 Innsbruck, Austria

**Keywords:** Gut microbiome, Spatial microbiome organization, Dysbiosis, Inflammation, Carcinogenesis, Fecal microbiome transplantation

## Abstract

The gastrointestinal (GI) tract harbors a diverse and host-specific gut microbial community. Whereas host-microbe interactions are based on homeostasis and mutualism, the microbiome also contributes to disease development. In this review, we summarize recent findings connecting the GI microbiome with GI disease. Starting with a description of biochemical factors shaping microbial compositions in each gut segment along the longitudinal axis, improved histological techniques enabling high resolution visualization of the spatial microbiome structure are highlighted. Subsequently, inflammatory and neoplastic diseases of the esophagus, stomach, and small and large intestines are discussed and the respective changes in microbiome compositions summarized. Finally, approaches aiming to restore disturbed microbiome compositions thereby promoting health are discussed.

## Introduction

All body surfaces being in contact with the environment, like the skin and the gastrointestinal (GI), urogenital, and respiratory tracts are colonized by microorganisms. These microbial consortia, collectively termed “microbiome” or “microbiota,” are now viewed as integral part of our body, being essential for proper organ function. Thus humans are considered holobionts composed of not only “own” (eukaryotic) cells but also microbial cells and understanding the mechanisms underlying health and disease development needs to encounter the microbial part of our body too [1, 2]. Different types of microorganisms are part of the human microbiome, including bacteria (prokaryotes), archea, fungi, protists, and virus. Dependent on the habitat, the composition of the microbiome differs significantly [3]. For instance, the gut microbiota is mainly populated by bacteria (> 99% [4]), whereas the skin harbors also significant amounts of fungi (≈ 10% [5]). The vast majority of commensal microorganisms reside in the colon, with estimates of up to 10^13^ bacteria followed by the skin, which harbors about 10^12^ bacteria. Thus, the collective microbiome colonizing our body (approx. 10^13^ microbial cells) outnumbers our own nucleated body cells (approx. 10^12^) by the factor of 10, which gives already an estimate of the biological potential of our “second genome” [4, 6].

The biological functions conferred by the microbiome are manifold. The gut microbiome is a major factor involved in metabolism and energy regulation [7]. Up to 10% of our daily consumed calories are provided by the gut microbiota partly via degradation of complex (plant-derived) polysaccharides into short-chain fatty acids (SCFAs; e.g., butyrate), a process called fermentation. Thus, the gut microbiome is a major factor contributing to obesity and its sequels like type II diabetes mellitus [8–10]. Another prominent feature of the microbiome is the education of the immune system. The mucosal immune system needs to tolerate the resident microbiome, whereas it needs to react against pathogens. This homeostasis is achieved by an intricate interplay of the microbiome and the host [11]. Especially the induction of tolerance via induction of anti-inflammatory cells and cytokines (e.g., regulatory T cells, IL-10, TGFβ) is an important trait of the microbiota, conferred by special microbial products directly interacting with the host’s immune system [12–15]. In addition, it has been recognized that CD4^+^ T cell responses are directed and modulated by specific commensals towards a T helper (Th) cell 1 or Th17 immune reaction, which has major implications not only in mucosal defense but also in autoimmune and autoinflammatory processes beyond the GI tract [16].

Moreover, physiologically colonized body surfaces are intrinsically protected from colonization with pathogens, a highly effective defense mechanism called pathogen exclusion or colonization resistance [17]. Various mechanisms have been described in this context. So it has been shown that a low-fiber diet quickly shapes the structural composition of the microbiome promoting the expansion of a mucus-degrading microbiota. This renders mice more susceptible to colitis elicited by certain intestinal pathogens [18]. On the other hand, a fiber-rich diet reduces the numbers of mucus-degrading commensals and promotes the bloom of fiber-degrading SCFA-producing bacteria. SCFA then support mucosal barrier functions through distinct mechanisms impacting on oxygen consumption by intestinal epithelial cells [19], modulating the threshold of intracellular danger receptors such as inflammasomes [20], or shifting naïve T helper cells towards regulatory T cells [13]. Other mechanisms include less-well investigated mechanisms of microbe-microbe interactions. So it has been shown that by producing iron-binding siderophores certain pathobionts and pathogens acquire a growth advantage during colitis when iron is scarce [21]. Certain protective commensals harness this circumstance by coupling these siderophores with antimicrobial microcins, which then enter and target pathobionts as “Trojan horses” through siderophore-receptor based uptake [22].

## Factors shaping the spatial organization of the human gut microbiome

Babies are born sterile. During birth, the body becomes immediately colonized by microbes from the surroundings, which is the main determinant shaping microbiome composition early in life [23]. Consequently, babies born naturally acquire different microbes like *Lactobacillus* and *Prevotella* resembling the mother’s vaginal microbiota, whereas babies born via Cesarian section are dominated by “skin”-type bacteria like *Staphylococcus*, *Corynebacterium*, and *Propionibacterium* [24]. Interestingly, differences in early colonization are supposed to contribute also to different susceptibilities to immune-mediated diseases, like asthma and allergies, later in life [25]. The first year of life is signified by an increased variability of the microbiome, which “stabilizes” when adult diet is introduced after weaning [26]. At this time-point (about 1 year of age), the infant microbiome resembles largely an adult microbiome.

The structure of the human microbiome is mainly determined by environmental factors like diet. Consequently, relatives or individuals living in the same household and having the same living habits share more microbes than unrelated individuals [27]. Overall, the gut microbiome appears to be quite stable over time for years, possibly life-long [26]. In addition, there seems to be also a (small) heritable component determining GI microbiome structure [28–30]. For instance, several genotyping studies correlating host genotype with gut microbiome composition have revealed a genetic association of the human lactase gene locus with *Bifidobacterium* abundance providing evidence of a gene-diet-gut microbiome interaction and giving new clues about pathogenesis of lactose intolerance [31, 32].

**Fig. 1:**
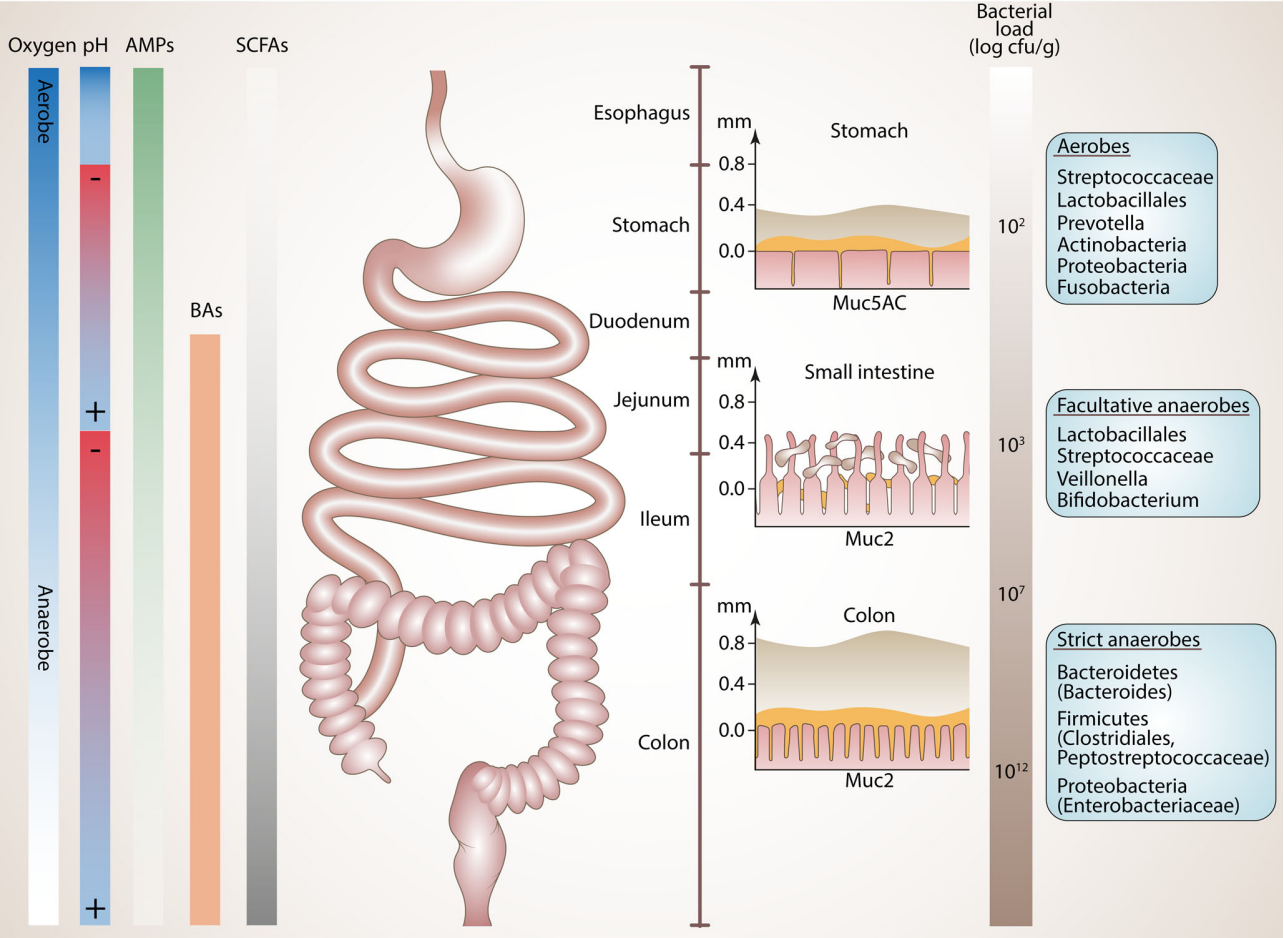
Biogeography and factors shaping the spatial organization of the gut microbiome. Left: factors determining gut segment specific microbiome composition like oxygen, pH, bile acids (BA), antimicrobial peptides (AMPs), and concentration of short-chain fatty acids (SCFAs). Middle: schematic representation of the GI tract and of the segment specific mucus layer architecture (adapted from [33, 34]). The inner solid (amber) and the outer loose mucus layer (gray) are shown. Note that in the stomach and colon the mucus layer is continuous, whereas in the small intestine the layer is discontinuous. Muc5AC and Muc2 denote the dominant mucins produced in the respective gut segment. Right: bacterial load and typical taxonomic compositions of different gut segments

The human GI tract could be simply seen as a tube with an input and output. A constant flux of microbes originating from the environment, diet, and the oral cavity exists which potentially facilitates entry of foreign and potentially harmful microbes. Nevertheless, a quite specific and stable microbiota is maintained in each gut segment under healthy conditions. Compositions and densities of the gut microbiome along the GI tract are mainly governed by biochemical factors like pH, oxygen, antimicrobial peptide (AMP) gradients, and presence of bile acids, as well as the speed of transit [33]. As indicated in Fig. 1, pH is lowest in the stomach, gradually increases towards the terminal ileum, decreases in the cecum, and again gradually increases towards the distal colon. An oxygen gradient exists along the length of the gut, with levels highest in the upper GI tract which decrease to anaerobic conditions in the distal colon. However, there is also a radial oxygen gradient in the colon, with anoxic conditions in the lumen and a slight increase in oxygen tension towards the mucosa, which can be consumed by microorganisms living in proximity to the mucosa, also from enteropathogens [35]. Each gut segment harbors a unique repertoire of AMPs, specifically suppressing certain groups of bacteria. Saliva contains high amounts of lysozyme efficiently degrading the murein wall of microbes and also the stomach epithelium is able to produce AMPs [36]. In the small intestine, Paneth cells secrete AMPs like α-defensins, C-type lectins, lysozyme, and phospholipase A2. In the large intestine, enterocytes secrete AMPs like β-defensins, C-type lectins, cathelicidins, galectins, and lipocalin [37]. Interestingly, the microbiome of the large intestine encodes genes providing resistance to specific AMPs conferring resilience to the microbiome during inflammation, when AMP levels are high, allowing faster recovery of a “healthy” microbiome after infection [38].

**Fig. 2:**
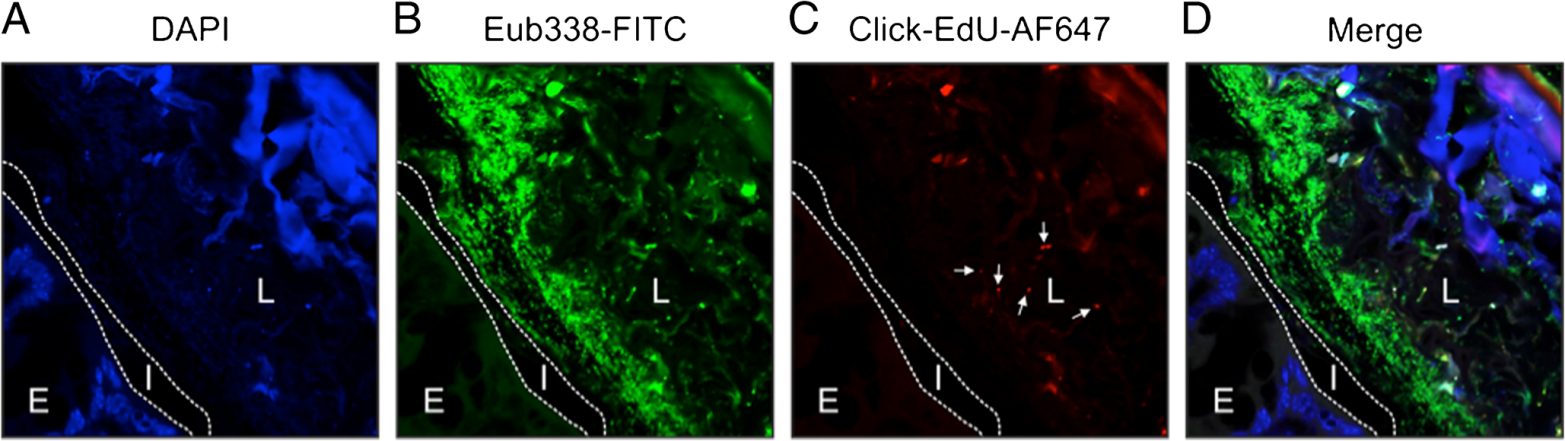
In vivo tracking and improved spatial resolution of gut-microbiome interactions. A mouse colon was fixed in Carnoy’s solution to preserve mucus layer architecture. **a** The section was counterstained with 4′,6-diamidino-2-phenylindole (DAPI) indicating the colonic epithelium (E) in the left lower corner, the interlaced (I) mucus layer (indicated by the two dotted lines), which is devoid of bacteria, and the colonic lumen (L) on the right side. The structure in the upper right depicts a plant component. **b** Bacteria were stained by FISH using a fluorescein isothiocyanate (FITC) pan-specific EUB338 probe (green) which covers approximately 90% of the domain bacteria. **c** In this experiment, mice were gavaged with the gut bacterium *Alistipes finegoldii* (phylum *Bacteroidetes*), which were cultured in the presence of the thymidine analog 5-ethynyl-2′-deoxyuridine (EdU). Metabolically active *Alistipes* was tracked utilizing click chemistry that is based on a copper-catalyzed covalent reaction between an alkyne (within the EdU) and an AlexaFluor® 647-containing azide. Note that the bacterium colonizes the luminal site of the colon, not the mucus layer (arrows). **d** The right picture shows the merged panels. Cells were imaged on a Zeiss Axioobserver Z1 microscope equipped with a LSM700 confocal unit. Original magnification 400×

The microbiota is spatially organized along the transverse axis of the GI tract, from the lumen to the mucosa. A major factor driving this transverse organization is the mucus layer covering the GI tract. Mucins are gel-forming glycoproteins, polymerizing into a mesh like structure. There are two discriminable mucus layers in the stomach and colon, an outer “loose” layer which is densely populated by bacteria and an inner “solid” layer, which is enriched in innate and adaptive immune effectors providing a biochemical barrier that segregates the microbiota from direct contact with the epithelium [34, 39]. This inner layer is nearly free of microbes. However, specific microbes like the mucin utilizer *Akkermansia muciniphila* are able to degrade the layer thus reaching inner areas [40, 41], in addition to various pathogens, like *Helicobacter pylori* in the stomach or enteropathogenic *Escherichia coli*, *Salmonella*, *Yersinia*, and *Campylobacter* in the colon. In the small intestine, the mucus layer is discontinuous and less defined; the tips of the villi often not covered by mucin (Fig. 1). Importantly, routine histological preparations do not preserve mucus layer architecture. The mucus layer is heavily hydrated and dehydration, which happens during conventional fixation with formaldehyde, shrinks the mucus layer leading to a very thin film lining the epithelium. Techniques for improved mucus preservation employ fixation of tissue with chloroform, dry methanol, and glacial acetic acid (i.e., Carnoy’s fixative), and processing in water-free solutions before embedding in paraffin, which preserves mucus layer architecture [34, 39, 42]. Moreover, this fixation method allows also for simultaneous staining of bacterial RNA/DNA by fluorescence in situ hybridization (FISH). Application of this technique allows for a high-resolution analysis of gut-microbiome interactions as exemplified in Fig. 2, wherein a mouse colon fixed in Carnoy’s solution was used to track a specific gut pathobiont (*Alistipes finegoldii*) in the GI tract in situ.

The total number of microbes increases from the esophagus to the distal colon, where the microbial load is estimated to be 10^12^ microbes per gram of feces. The acidic pH and oxygenated environment of the upper GI tract limit microbial colonization to acid- and oxygen-tolerant bacteria (e.g., *Lactobacillus*, *Streptococcus*, *Veillonella*), whereas in the large intestine the flow is slower, and metabolism favors fermentation of complex plant-derived polysaccharides (e.g., fiber) or from host mucus. This results in greater species richness (i.e., number of prevalent taxa), higher microbial densities and dominance of the saccharolytic *Bacteroidales* and *Clostridiales* in the large intestine [39].

## Dysbiosis—chicken and egg

It is important to note that many studies investigating the microbiome in the disease context describe associations and the reported shifts in microbiome composition (termed “dysbiosis”) are often not proven causal for the respective disease and could just represent epiphenomena, wherein changed habitat factors (e.g., by change of substances produced by the host cells during disease) lead to altered microbial community compositions [43]. However, changed habitat factors and subsequent dysbiosis could contribute to disease as well, especially if these alterations are persistent. Paradigmatic in this context are epithelial tumors of microbially colonized organs like colorectal cancer (CRC) and its precursors (adenomas). Recently, it has been shown that the neoplastic colon epithelium overexpresses a polysaccharide, D-galactose-b(1-3)-N-acetyl-D-galactosamine (Gal-GalNAc), which is selectively bound by a *Fusobacterium nucleatum* lectin (Fap2) enriching this protumorigenic microbe in CRC tissue [44]. Another impressive example for a direct contribution of the microbiome to disease pathogenesis is exemplified by approaches aiming in restoration of dysbiosis, like bacteriotherapy or fecal microbiome transplantation (FMT). This approach is already regularly used to treat recurrent *Clostridium difficile* (pseudomembranous) colitis a disease caused by antibiotic-induced dysbiosis and subsequent pathogen overgrowth [45, 46]. Importantly, the restoration of dysbiosis by FMT shows also efficacy in chronic inflammatory GI diseases like ulcerative colitis and even in metabolic diseases like in individuals with metabolic syndrome [47, 48].

## Microbiome and inflammatory diseases of the upper GI tract

### Esophagus and gastro-esophageal junction

The composition of the esophageal microbiome is heavily influenced by microbes originating from the oral cavity, dominated by taxa like *Streptococcus* followed by *Prevotella*, *Veillonella*, and *Fusobacterium*, which represent the healthy esophageal core microbiome [49–51]. Chronic exposure of the distal esophagus to gastric acid and duodenal bile salts is thought to be a major factor underlying the pathogenesis of gastro-esophageal reflux disease (GERD), Barrett’s esophagus (BE), and subsequently adenocarcinoma of the gastro-esophageal (GE) junction. As discussed above, changing habitat factors lead also to altered microbiome compositions, which could in turn fuel inflammation and tumorigenesis. Certain studies noted significant taxonomic changes in the esophageal microbiome in GERD, BE, and adenocarcinoma of the GE junction. Dominant taxa like the Gram-positive *Streptococcus* are depleted, whereas Gram-negative taxa like *Veillonella*, *Prevotella*, *Campylobacter*, *Fusobacterium*, *Haemophilus*, and *Neisseria* are enriched in the diseased states [52, 53]. Notably, the oral taxons *Campylobacter concisus* and *C. rectus* have been found enriched in the mucosa of GERD and BE but were depleted in adenocarcinoma of the GE junction [54, 55]. Interestingly, *C. concisus* seems to be adapted to the harsh (acidic) environment of the upper GI tract. Significantly increased RNA transcripts of *C. concisus* compared to other stomach microbes were detected in the human gastric juice [56]. These taxa are also increased in individuals with IBD, especially children with Crohn’s disease [57]. In addition, also *Fusobacterium nucleatum*, a Gram-negative filiform bacterium normally inhabiting the human oral cavity (dental plaque), is overabundant in Crohn’s disease, in addition to its association with adenomas and CRC [58]. Importantly, several molecular studies have shown a proinflammatory and protumorigenic behavior of *F. nucleatum*, wherein the bacterium was shown to specifically activate epithelial cell proliferation, to induce a protumorigenic immune-microenvironment and inhibits immunological tumor surveillance [59–61].

Another prominent disease of the esophagus is represented by eosinophilic esophagitis (EoE). Although the primary cause of EoE is thought to be a non-IgE-mediated food hypersensitivity [62], also increased levels of Gram-negative bacteria (*Neisseria*, *Corynebacterium*, *Haemophilus*) were reported [63, 64]. If these microbiome changes also contribute to disease pathogenesis needs to be clarified. Recent reviews have summarized the majority of existing studies investigating microbiome compositions in diseases of the upper GI tract [49–51].

### Stomach

**Fig. 3:**
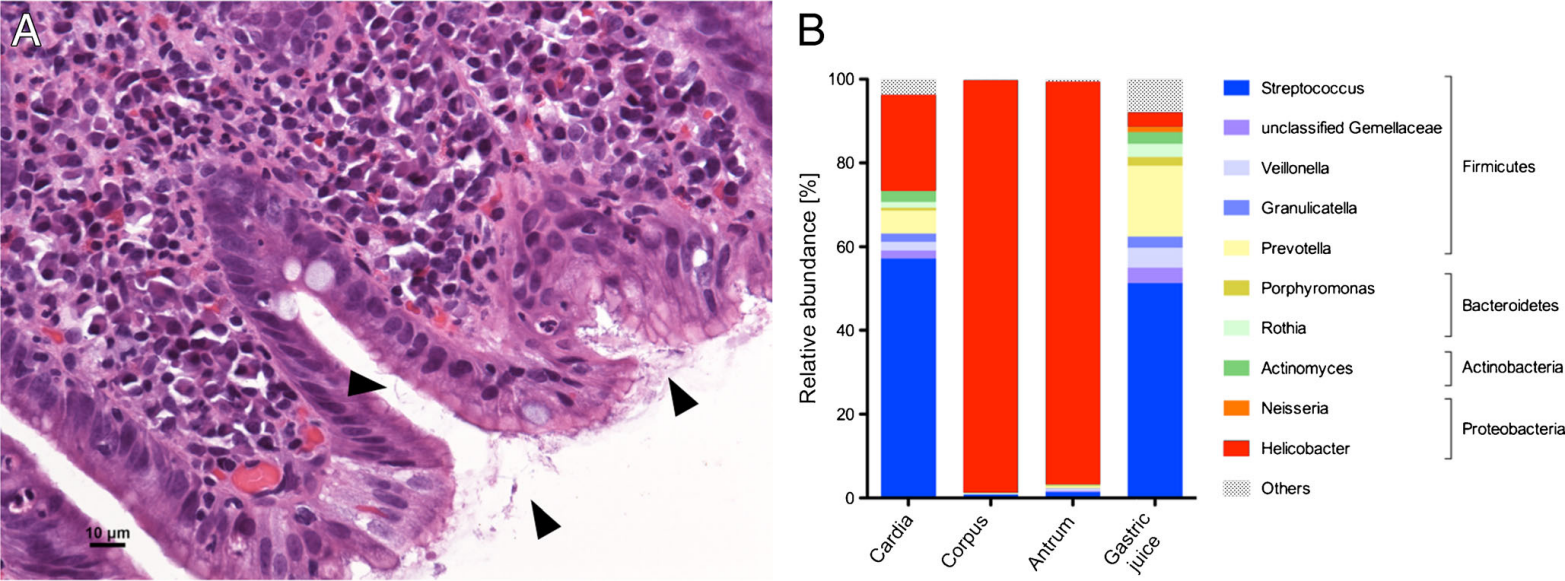
Stomach microbiome in chronic *H. pylori* gastritis. **a** Gastric corpus biopsy signifying the preferred mucosal niche of *H. pylori* (arrow heads). **b** Microbiome analysis (based on the 16S rRNA gene) indicates the dominance of *H. pylori* at the mucosal sites, whereas in gastric juice only few *H. pylori* are prevalent

*Helicobacter pylori* is a bacterium purely adapted to the human host. This restricted host spectrum has led to a coevolution of the bacterium with humans [65] and has shaped the molecular determinants of host-pathogen interaction manifold [66]. Noteworthy, the intimate relationship between the bacterium and humans has led also to beneficial effects of *H. pylori* infection aside of the clear pathogenic effects leading to chronic gastritis, ulcers, and subsequently gastric adenocarcinoma and MALT lymphoma [67, 68]. Noteworthy, it has for instance been shown that early life-time infection with *H. pylori* lowers significantly the risk of developing asthma and celiac disease later in life [69, 70]. Many of this beneficial traits are induced by immune system modulation of *H. pylori*, due to the induction of a tolerogenic immune-state (e.g., induction of regulatory T cells), which helps the bacterium to persist in the human host. Therefore, *H. pylori* represents a paradigm “pathobiont,” a term which specifies bacteria with a commensal and pathogenic lifestyle that is determined not just exclusively by bacterial traits but also by host (e.g., age, concomitant microbiome) or environmental factors [71]. The view of a commensal lifestyle of *H. pylori* is also supported by reports showing low level colonization of asymptomatic individuals [72–76]. Under disease conditions, *H. pylori* is the dominant stomach bacterium outcompeting the normal resident microbiome and its preferred niche is the gastric mucosal surface (Fig. 3). Interestingly, *H. pylori* infection also significantly impacts lower gut microbiome composition [77, 78]. That pathogenicity of *H. pylori* is also determined by non-*H. pylori* factors is supported by the finding that the concomitant gastric microbiota is also important to drive gastric tumorigenesis. Interestingly, distinct sequential changes in microbiome compositions occur along the gastric metaplasia-dysplasia development [79–81]. In analogy to adenocarcinoma of the GE junction also bacteria originating from the oral cavity seem to be involved in tumorigenesis of the *H. pylori*-infected stomach [82].

Another form of chronic gastritis is lymphocytic gastritis (LyG), characterized by increased CD8^+^ intraepithelial lymphocytes (IELs; ≥ 25 per 100 epithelial cells). In addition to the association with celiac diseases (CeD), a great proportion of LyG has unclarified causes. *H. pylori* is normally absent in LyG; however, eradication therapy seems to be an effective treatment of LyG, even in the absence of identifiable *H. pylori*, suggesting an alternative bacterial cause for the disease. We recently identified *Propionibacterium acnes* as a possible LyG disease trigger inducing the natural killer group 2 member D (NKG2D) system and the proinflammatory cytokine interleukin (IL)-15 in the gastric mucosa [76]. Natural killer (NK) cells, CD8^+^ T cells, and certain other T cells express the NKG2D receptor. The NKG2D receptor ligands (NKG2DLs) are expressed mainly on epithelial cells at low levels under physiological conditions, but their expression is induced under conditions of cell stress, such as infection, neoplastic transformation, or challenge with specific metabolites (e.g., short-chain fatty acids, SCFAs) or gliadin in the case of CeD. Upon ligand-receptor interaction, NKG2D triggers a cytotoxic response in the receptor-bearing lymphocyte, eliminating the stressed cell that is overexpressing the ligand. This reaction is enhanced by the presence of IL-15 [83–86]. Of note, the NKG2D-NKG2DL system and IL-15 are important for immunological tumor surveillance, which is necessary for the elimination of neoplastic cells. The system has therefore been investigated as a potent target for cancer immunotherapy [87, 88].

### Small intestine

In CeD, the NKG2D system is critical for recruitment of CD8^+^ IELs and subsequent villus atrophy in the duodenum [83, 84]. In addition to the genetic causes (i.e., HLA-DQ), also environmental factors play a role in the development of CeD, including the microbiome [89]. Phenomena like the so-called Swedish CeD epidemic, wherein the incidence of CeD fourfold increased in children within a short period followed by a rapid drop, resembles an infectious disease (i.e., “out-break pattern”) [90]. Recently, it was shown that gut bacteria are able to differentially degrade gluten giving a possible explanation for these phenomena. Specifically, overgrowth of the opportunistic pathogen *Pseudomonas aeruginosa* was reported in CeD, which is able to produce a specific elastase (lasB). This enzyme degrades gluten in a specific manner enabling the released peptides to better translocate the intestinal barrier, which subsequently leads to the activation of gluten-specific T cells driving the disease [91].

Another inflammatory disease often prevalent in the small intestine, wherein the microbiome plays a role, is graft versus host disease (GvHD). GvHD is caused by the alloactivation of T cells, which recognize host antigens as foreign, causing autoimmune attack to organs such as the GI tract, lungs, liver, and skin [92]. A microbial factor contributing to disease development was already suspected in the 1970s when it was demonstrated that mice kept under germ-free conditions developed less GI GvHD [93, 94]. Although the underlying molecular mechanisms of the contribution of the microbiome to GvHD development are largely unknown, depletion of the resident microbiome, due to the intensive antibiotic and chemotherapeutic treatment regimens, seems to play a pivotal role. Notably, the magnitude of intestinal diversity loss is a risk factor for treatment-related mortality including death from GvHD [95]. The intestinal diversity loss (e.g., depletion of specific *Clostridia*) leads to impaired microbial fermentation and a lack of SCFAs (e.g., butyrate), the main energy source of gut epithelia. SCFA deprivation has been shown to induce apoptosis in intestinal epithelial cells, the hallmark histological change observed in GvHD [96]. Moreover, a common dysbiotic fecal microbiome signature in GvHD was reported recently, specified by an (over-)abundance of *Enterococcus* species (*E. faecium*, *E. faecalis*). This microbiome type is significantly associated with the risk to develop gut GvHD after hematopoietic stem cell transplantation [97]. Interestingly, treatment strategies which restore a physiological gut microbiome (e.g., FMT) appear to be beneficial in patients with chronic active GvHD [92, 98].

## Microbiome and inflammatory diseases of the lower GI tract

### Antibiotic-associated colitis

**Fig. 4:**
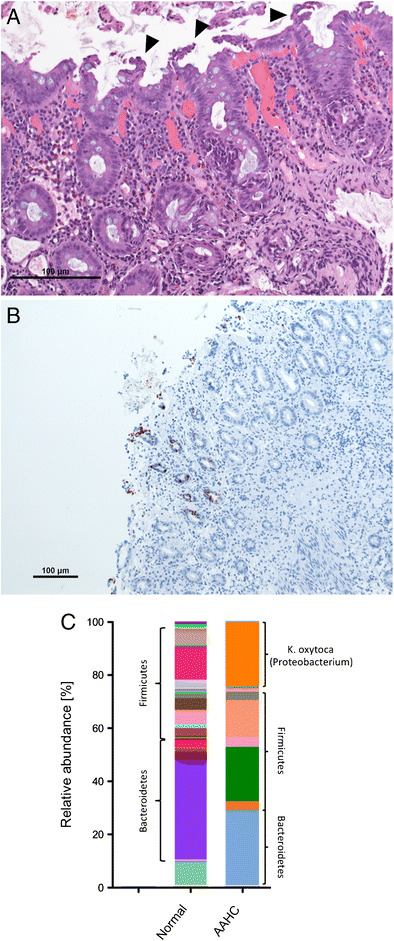
Histology and microbiome representation of antibiotic-associated hemorrhagic colitis (AAHC). **a** Colon histology with micropapillary epithelial protrusions (arrow heads) indicating the cytotoxic effect of the enterotoxin tilivalline produced by *K. oxytoca*. **b** Activated caspase-3 immunohistochemistry signifying epithelial cell apoptosis. **c** Fecal microbiome composition in AAHC (based on the 16S rRNA gene analysis). A highly reduced overall diversity is evident with the overgrowth of the proteobacterium *K. oxytoca*. A diverse healthy fecal microbiome is shown on the left

Colitis is a frequent side effect of antibiotic therapy [99]. Besides direct drug-induced toxicity of antibiotics, depletion of the gut microbiome and subsequent pathogen overgrowth are major disease causes, like in *Clostridium difficile* colitis (CDC [45]). In CDC, the bile acid 7α-dehydroxylating intestinal bacterium *Clostridium scidens* seems to be depleted which leads to a lack of suppression of *C. difficile* [100]. Noteworthy, FMT is already an established highly effective treatment for recurrent CDC, indicating the potential of therapies aiming to restore an altered microbiome [46]. Of note, antibiotic-associated colitis could be caused also by other pathogens, like *Klebsiella oxytoca*, the causative agent of antibiotic-associated hemorrhagic colitis (AAHC). AAHC is usually observed after therapy with penicillins and represents as segmental, often patchy hemorrhagic colitis, typically dominant in the right colon [101, 102]. In AAHC, overgrowing *K. oxytoca* intrinsically resistant to beta-lactams and producing the enterotoxin tilivalline lead to intestinal epithelial apoptosis and colitis (Fig. 4). In extreme forms of antibiotic-associated colitis microbiome depletion can lead to disease courses resembling severe acute GvHD. We described recently a series of severe apoptotic enterocolitis cases emerging after therapy with antibiotics and steroids, wherein severe microbiome depletion seemed to trigger the disease. Notably, FMT performed in one case restored a normal gut microbiome and was highly effective to dampen epithelial cell death and enterocolitis [103].

### Inflammatory bowel diseases

Crohn’s disease and ulcerative colitis represent the two major clinically defined forms of inflammatory bowel diseases (IBD). Our current mechanistic understanding puts the intestinal epithelial cell as the central orchestrator of the innate immune system into the limelight, integrating genetically based interactions between the intestinal microbiome, the mucosal immune system, and environmental factors [104]. A genetic component in IBD has been postulated early in the process of identifying and understanding disease etiology with evidence from early epidemiological [105, 106] and twin studies [107]. Recent genome-wide association studies (GWAS) identified more than 200 IBD susceptibility loci, with most of them conferring modest disease risk in terms of odds-ratios [108]. Nevertheless GWAS resulted in new insights in IBD biology revealing a substantial overlap between the genetic risks of CD and UC and between other autoimmune and autoinflammatory diseases [109, 110]. Furthermore, these techniques pointed out previously unappreciated pathways in IBD, ahead of autophagy [111].

Nevertheless, the incidence of both Crohn’s disease and ulcerative colitis is increasing dramatically worldwide, which is of course hardly explained by changes in the genetic landscape [112]. IBD was among the first diseases in which the microbiome has been studied intensively. These studies identified not only significant compositional alterations of the microbiome with reduction of potentially-protective commensals such as *Faecalibacterium prausnitzii* [113] but also a less stable microbiome, an increased adherence of microbes to the epithelial surface, however, no clear signature with specific pathobionts. Of note, many of the early studies included patients under medication which potentially influenced some of the results. More recently, Gevers and colleagues studied the treatment-naïve microbiome including 447 children and adolescents with newly diagnosed Crohn’s disease and 221 controls. They identified a strong correlation of disease with increased abundance of *Enterobacteriaceae*, *Pasteurellacaea*, *Veillonellaceae*, and *Fusobacteriaceae* and a decreased abundance of *Erysipelotrichales*, *Bacteroidales*, and *Clostridiales* [58]. This study included several other interesting aspects showing that composition of the gut microbiome may be predictive for an individual disease course and that in early-stage and lower grade inflammation the mucosa-associated microbiome, e.g., from rectal biopsies, may be superior to fecal microbiome analysis. Noteworthy, more recent studies implied relevant alterations in the structural composition of the gut “virome” in IBD [114], and two recent clinical trials showed that blocking IL-17A with secukinumab or IL-17RA with brodalumab worsened Crohn’s disease, with some patients developing mucocutaneous candidiasis ventilating a role for the gut mycobiome in IBD, which has been implicated in intestinal inflammation as well [115, 116].

Are aberrant immune responses to the intestinal microbiome indeed causally related to IBD? Evidence from clinical studies showed that mucosal inflammation recurs quickly after reinfusion of ileal content from a protective proximal loop ileostomy, which certainly proved that the “enemy” lies within the “fecal stream” [117]. Various experimental models implicated that an altered dysbiotic microbiota causes transmissible disease, both in the ileum and the colon. Schaubeck and coworkers demonstrated that only a certain proportion of TNF^ΔARE^ mice, which overproduce TNFα and develop spontaneous ileitis, will develop high-grade histological inflammation, while others show no inflammation at all, despite identical genetic background and a comparable environment. Using a reductionist approach, they transplanted the stool from inflamed and non-inflamed TNF^ΔARE^ mice into germ-free animals and demonstrated that the microbiome was indeed the driving force for inflammation in this model [118]. Our own studies indicated a transplantable dysbiotic microbiome in mice double-deficient of IL-10 and lipocalin 2 (Lcn2) driving colonic inflammation. In cross-foster experiments, IL-10 pups raised by double-deficient nursing mothers developed the same phenotype irrespectively of their own genotype [119].

However, there are additional relevant environmental modulators of the intestinal microbiome. “We are, what we eat” and the axis food-microbiome represents another important player particularly in IBD [120]. From clinical trials, we have learned that an elemental or polymeric diet is highly effective in the treatment of Crohn’s disease in children [121]; however, the underlying mechanisms remain elusive as recent studies suggest that an exclusive enteral nutrition further aggravates dysbiosis [122]. Moreover, a western diet, enriched in animal protein and fat, reduced in dietary fiber, and the intake of processed foods has been shown to promote intestinal inflammation through different mechanisms [15, 123, 124].

### Colorectal cancer

Colorectal cancer (CRC) represents the third most commonly diagnosed malignancy and the fourth leading cause of cancer-related deaths worldwide. CRC burden is expected to increase by 60% until 2030. CRC is one of the clearest markers of cancer transition and incidence is growing fastest in low- and middle-income countries and is associated with adoption of a western lifestyle [125]. Mechanistically, malignant transformation of intestinal epithelial cells and the development of CRC includes at least three relevant steps namely (i) the induction of oncogenic mutations within the Lgr5+ intestinal stem cells (SC), (ii) an altered beta-catenin/Wnt signaling, and (iii) proinflammatory cascades such as TNFα-NFκB and IL6-STAT3 that catalyze CRC development [126].

CRC has been associated with specific changes in gut microbiome composition [127–129]. Recently, we studied microbial alterations along the adenoma-carcinoma sequence, collecting stools from healthy controls, patients with advanced adenomas and patients with CRC [127]. A metagenome-wide association study (MGWAS) was performed and found, that certain *Bacteroides* spp. (e.g., *B. dorei*, *B. vulgatus*, *B. massilensis*) and *E. coli* were associated with systemic inflammation and tumor stage. In line with others, *Parvimonas*, *Bilophila wadsworthia*, *Fusobacterium nucleatum*, and *Alistipes* spp. were also overrepresented in CRC patients. Importantly, the presence of SCFA-producing and bile acid-metabolizing bacteria was clearly positively related to consumption of meat and negatively related to the intake of fruits and vegetables again indicating the important role of a western lifestyle in development of CRC [127].

There is emerging evidence regarding a causal role of certain bacteria in CRC evolution such as *F. nucleatum*, colibactin-producing *E. coli* and toxigenic *Bacteroidis fragilis*. Interestingly, little is known about bacteria and mechanisms that protect from CRC development. *F. nucleatum* was one of first bacteria associated with human CRC, shown to be enriched in tumors [59, 130]. Furthermore, *F. nucleatum* has been strongly associated with certain tumor types, such as microsatellite instable CRC and cancers with BRAF mutations [131, 132]. Mechanistically, it has been shown that the *F. nucleatum* FadA antigen is a ligand for E-cadherin on intestinal epithelial cells that activates the β-catenin signaling pathway, thereby promoting uncontrolled cell growth, acquisition of a stem cell-like phenotype of epithelia and loss of cell polarity [61]. Also, mucosa-associated *E. coli* are overrepresented in CRC, which correlates with tumor stage and prognosis [133]. Moreover, some *E. coli* strains harbor a genomic polyketide synthase (*pks*) island that encodes for the genotoxin colibactin capable of inducing DNA damage and mutations in epithelial cells [134].

Finally, it is increasingly recognized that the microbiome contributes to the efficacy of cancer therapies. Several recent papers demonstrated convincingly that bacterial nucleotide metabolism genes affect efficacy of 5-fluoruracil and camptothecin antineoplastic therapy [135]. Again, *F. nucleatum* promoted colorectal cancer resistance to chemotherapy by a complex network of mechanisms including toll-like receptor signaling, microRNAs and induction of autophagy [136]. The intestinal microbiome and associated intestinal immune mechanisms seem particularly relevant for response to checkpoint inhibitors such as anti-PD1 and anti-PDL1 therapies. This was first recognized in experimental models, wherein response to anti-PD-L1 antibodies was associated with the presence of *Bifidobacterium* and oral administration of *Bifidobacterium* boosted the efficacy of such therapies [137]. Gopalakrishnan and coworkers recently demonstrated that the efficacy of anti-PD1 therapy was strongly affected by concomitant antibiotic therapy, as well. Mechanistically, the response was strongly dependent on the presence of the mucin degrader *A. muciniphila*. Response to anti-PD1 therapy in mice that received the stool from non-responders could be restored by administration of *A. muciniphila* and was dependent on *Akkermansia*-induced, IL-12-dependent Th1 responses [138].

## Conclusion

It is now evident that the human gut microbiome significantly contributes not only to the maintenance of GI health but also to disease development. Recent scientific findings support the view that the gut microbiome might serve as a future diagnostic and therapeutic target for inflammatory and also neoplastic GI diseases.
